# The Myocardial Ischemia Evaluated by Real-Time Contrast Echocardiography May Predict the Response to Cardiac Resynchronization Therapy: A Large Animal Study

**DOI:** 10.1371/journal.pone.0113992

**Published:** 2014-12-03

**Authors:** Yongle Chen, Leilei Cheng, Haohua Yao, Haiyan Chen, Yongshi Wang, Weipeng Zhao, Cuizhen Pan, Xianhong Shu

**Affiliations:** Department of Echocardiography, Zhongshan Hospital, Fudan University, Shanghai Institute of Cardiovascular Diseases, Shanghai Institute of Medical Imaging, Shanghai, PR China; Georgia Regents University, United States of America

## Abstract

Evidence-based criteria for applying cardiac resynchronization therapy (CRT) in patients with ischemic cardiomyopathy are still scarce. The aim of the present study was to evaluate the predictive value of real-time myocardial contrast echocardiography (RT-MCE) in a preclinical canine model of ischemic cardiomyopathy who received CRT. Ischemic cardiomyopathy was produced by ligating the first diagonal branch in 20 beagles. Dogs were subsequently divided into two groups that were either treated with bi-ventricular pacing (CRT group) or left untreated (control group). RT-MCE was performed at baseline, before CRT, and 4 weeks after CRT. Two-dimensional speckle tracking imaging was used to evaluate the standard deviation of circumferential (Cir12SD), radial (R12SD), and longitudinal (L12SD) strains of left ventricular segments at basal as well as middle levels. Four weeks later, the Cir12SD, R12SD, and myocardial blood flow (MBF) of the treated group were significantly improved compared to their non-CRT counterparts. Furthermore, MBF values measured before CRT were significantly higher in responders than in non-responders to bi-ventricular pacing. Meanwhile, no significant differences were observed between the responder and non-responder groups in terms of Cir12SD, R12SD, and L12SD. A high degree of correlation was found between MBF values before CRT and LVEF after CRT. When MBF value>24.9 dB/s was defined as a cut-off point before CRT, the sensitivity and specificity of RT-MCE in predicting the response to CRT were 83.3% and 100%, respectively. Besides, MBF values increased significantly in the CRT group compared with the control group after 4 weeks of pacing (49.8±15.5 dB/s vs. 28.5±4.6 dB/s, *p*<0.05). Therefore, we considered that myocardial perfusion may be superior to standard metrics of LV synchrony in selecting appropriate candidates for CRT. In addition, CRT can improve myocardial perfusion in addition to cardiac synchrony, especially in the setting of ischemic cardiomyopathy.

## Introduction

Heart failure, a complex clinical syndrome that encompasses a host of abnormalities in cardiac structure and function, is a major public health epidemic that accounts for a high degree of mortality and morbidity worldwide [1]. Beyond routine oral medication, cardiac resynchronization therapy (CRT) is a viable treatment strategy for patients with refractory heart failure [2]. CRT by means of bi-ventricular pacing can support ventricular contraction, increase cardiac output, relieve heart failure symptoms, improve quality of life of patients, and reduce their overall mortality. Despite these highly encouraging effects, the overall efficacy of CRT is hampered by the fact that approximately 30% of patients who are administered the therapy do not benefit from bi-ventricular pacing and in some cases may exhibit worsening of their heart failure symptoms [3]–[5]. Although ischemic cardiomyopathy is clearly one of the most common heart failure etiologies, evidence-based criteria for applying CRT in patients with ischemic cardiomyopathy are still scarce.

Two-dimensional speckle tracking imaging (2D-STI) is a novel technique for evaluating cardiac motion after CRT [6]. Real-time myocardial contrast echocardiography (RT-MCE) has been used *in vivo* for decades to quantitatively assess myocardial perfusion by calculating myocardial blood flow (MBF). Accordingly, the aim of the present animal study was twofold: (1) to validate that changes in myocardial perfusion may be key factors in predicting the outcome of CRT in the setting of ischemic cardiomyopathy and (2) to explore that CRT can improve myocardial perfusion in this common disease etiology.

## Methods

### Canine model of ischemic cardiomyopathy

All protocols were approved by the Animal Care and Use Committee of Zhongshan Hospital of Fudan University. All surgery was performed under sodium pentobarbital anesthesia, and all efforts were made to minimize suffering.

Totally 20 adult female beagles were divided into two groups that either underwent bi-ventricular pacing (CRT group, n = 10) or did not (control group, n = 10). All the animals were anesthetized (3% sodium pentobarbital, 30 mg/kg) and intubated. Their chests were opened allowing ligation of their first diagonal branch, resulting in myocardial infarction (MI). An epicardial lead (CAPSURE EPI 4965, Medtronic, USA) was sutured to the surface of the marginal area of the MI. All beagles were followed for 2 weeks to document the presence and severity of heart failure after the MI procedure. Right atrial (MEMBRANE EX 1474K, Medtronic, USA) and ventricular leads (CAPSURE SENSE 4074, Medtronic, USA) were subsequently implanted along with the pacemaker unit (INSYNC III 8042, Medtronic, USA); meanwhile, the epicardial lead was connected to the pacemaker. Pacing was initiated in the CRT group only. After 4 weeks of pacing, animals receiving CRT were further divided into two subgroups on the basis of their response to CRT. The left ventricular ejection fraction (LVEF) of dogs in the control group was spontaneously improved after MI and peaked at 45% by 4 weeks after the intervention. Thus, LVEF ≥45% was considered a positive response to CRT after 4 weeks of resynchronization therapy in the CRT group. Animals in CRT group were then classified as “responders” or “non-responders” to the bi-ventricular treatment depending on their hemodynamic function after 4 weeks.

### Echocardiography protocol

The commercially available echocardiographic systems (Philips IE 33, Philips Medical Systems Corporation, MA, USA) was used, equipped with a 1–5 MHz transducer (S5-1) and a 1–3 MHz transducer (X3-1), by two experienced cardiologists. At baseline, 2 weeks after MI (before CRT), and 4 weeks after CRT was initiated, all beagles underwent two-dimensional, three-dimensional echocardiography as well as RT-MCE measurements. The data were stored in DICOM format. Furthermore, all the images were analyzed using Philips Q-Lab 8.1 work station (Philips Medical Systems Corporation, MA, USA).

The LVEF, left ventricular end diastolic volume (LVEDV), and left ventricular end systolic volume (LVESV) were obtained from three-dimensional images of apical four-chamber views. Left ventricular synchrony was quantified with 2D-STI, which were traced and analyzed automatically from apical 4-, 3-, and 2-chamber views (longitudinal strain), as well as the parasternal short axis views (radial and circumferential strain). The standard deviations of the time-to-peak of the transmural regional circumferential strain (Cir12SD), radial strain (R12SD), and longitudinal strain (L12SD) for 12 segments of basal as well as middle level of left ventricle were determined.

For RT-MCE, the contrast agent applied was the commercially available SonoVue (Brocco, Milan, Italy). The contrast agent 59 mg SonoVue was diluted in 5 ml saline and injected via anterior auricular veins slowly (2.5 mL per 1 mL/min). When imaging performed, the focus was initially set at two-thirds of the depth of the image and then moved at the level of myocardial segment to be examined. Mechanical index was set at 1.7 for flash images and 0.1 for real-time images. The definitive setting of the echocardiographic images was optimized after initial infusion, kept constant throughout the study, and matched at follow-up MCE study. At least 15 consecutive heart beats of every destruction-replenishment sequence of basal and middle views at short-axis of left ventricle were captured in disc for subsequent off-line analysis. The MBF value, a reliable measure of myocardial perfusion, was calculated from the product of the plateau video intensity (A) and the rate constant rise of the plateau video intensity (β), as previously reported [7].

### Statistical analysis

All results were summarized as mean ± SD. The independent Student’s *t* test was used for comparing results of the CRT and control groups. The paired Student’s *t* test was used for comparing changes within each group. A Pearson correlation coefficient was used in testing the relationship between MBF and LVEF after CRT. Results were considered statistically significant for two-sided *p*<0.05. All data were analyzed using SPSS 16.0 software (SPSS Inc, Chicago, IL, USA).

## Results

### Baseline characters of the canine model

Two dogs of CRT group and one dog of control group died for ventricular fibrillation following the surgical procedure. All other canine successfully completed the study (CRT group, 8 dogs; control group, 9 dogs). Since the LVEF of dogs in the control group was spontaneously improved after MI and peaked at 45% by 4 weeks after the intervention, we defined LVEF ≥45% was a positive response to CRT for the beagles in the bi-ventricular pacing group. Then, animals in CRT group were classified as “responders” or “non-responders” depending on their hemodynamic function after 4 weeks of resynchronization treatment. Accordingly, 2 of 8 dogs in the CRT group showed no significant response to CRT and were therefore classified as non-responders, whereas the other 6 dogs exhibited significant benefits.

As compared, there was no significant difference in LVEF, LVEDV, LVESV, MBF, Cir12SD, R12SD, or L12SD between the CRT and control groups at baseline and following MI (i.e., before CRT treatment; Table 1).

**Table 1:** The echocardiographic parameters of the two groups at baseline, before, and after CRT.

	control group (n = 9)			CRT group (n = 8)		
	Baseline	Before CRT	4 w after CRT	Baseline	Before CRT	4 w after CRT
LVEF (%)	71.9±6.1	30.0±3.0	39.0±5.9	73.0±5.9	31.9±4.4	53.3±12.6* †
LVEDV (ml)	26.8±3.5	34.6±2.7	34.0±2.1	27.1±3.8	35.3±2.1	30.0±4.1* †
LVESV (ml)	9.6±2.6	22.0±4.0	22.8±4.3	9.5±3.7	18.2±3.8	14.3±2.5* †
Cir12SD (ms)	19.7±3.5	44.2±9.7	41.7±10.8	18.8±3.3	40.8±5.4	31.2±6.9* †
R12SD (ms)	25.9±5.0	70.3±12.6	65.7±9.7	29.3±4.9	78.1±15.1	43.9±17.1* †
L12SD (ms)	31.3±9.9	55.3±18.0	55.9±15.0	35.6±7.6	59.4±16.1	55.4±9.7
MBF (dB/s)	76.4±9.3	23.6±5.7	28.5±4.6	70.2±11.3	25.9±5.9	49.8±15.5* †

*p<0.05 compared to that before CRT;

† *p*<0.05 compared to control group.

### Comparison of LVEF, LVEDV, and LVESV between the two groups after CRT

LVEF showed a greater significant increase in the CRT group (from 31.9% to 53.3%) than in the control group by 4 weeks after the initiation of bi-ventricular pacing in the CRT group (*p*<0.05). Similarly, animals in the CRT group exhibited a significantly greater reduction in LVEDV than those in the control group (*p*<0.05). As shown in Table 1, changes in LVESV were also consistent with greater improvement in the CRT group (*p*<0.05), thereby explaining the selective improvement in LVEF.

### Comparison of left ventricular synchrony index between the two groups after CRT

**Figure 1:**
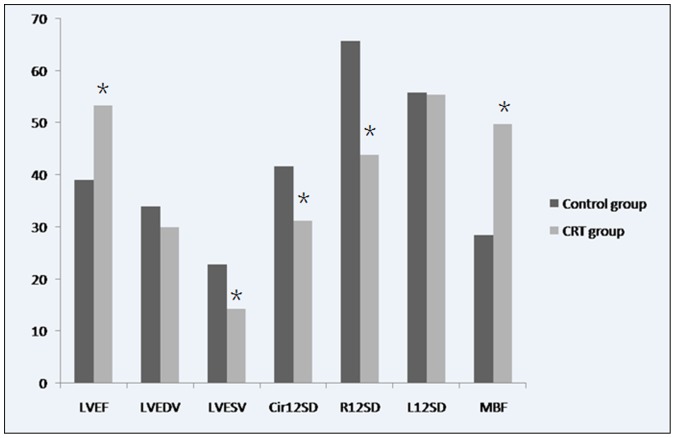
An illustration of LVEF, LVEDV, LVESV, Cir12SD, R12SD, L12SD, and MBF between control group and CRT group at 4 weeks after CRT. The LVEF, LVESV, Cir12SD, R12SD, and MBF were improved in CRT group. LVEF = left ventricular ejection fraction; LVEDV = left ventricular end diastolic volume; LVESV = left ventricular end systolic volume; Cir12SD = standard deviation of the time-to-peak of the transmural regional circumferential strain; R12SD = standard deviation of the time-to-peak of the transmural regional radial strain; L12SD = standard deviation of the time-to-peak of the transmural regional longitudinal strain; MBF = myocardial blood flow.

No significant differences in L12SD were observed between the groups before or after the intervention. In contrast, significant differences in Cir12SD and R12SD were found in the CRT group before and after bi-ventricular pacing (40.8 vs. 31.2 ms and 78.1 vs. 43.9 ms, respectively, all *p*<0.05). Furthermore, both Cir12SD and R12SD were more significantly improved in the CRT group compared to the control group (31.2 vs. 41.7 ms and 43.9 vs. 65.7 ms, respectively, all *p*<0.05; Table 1, Figure 1).

### Comparison of MBF between the two groups

**Figure 2:**
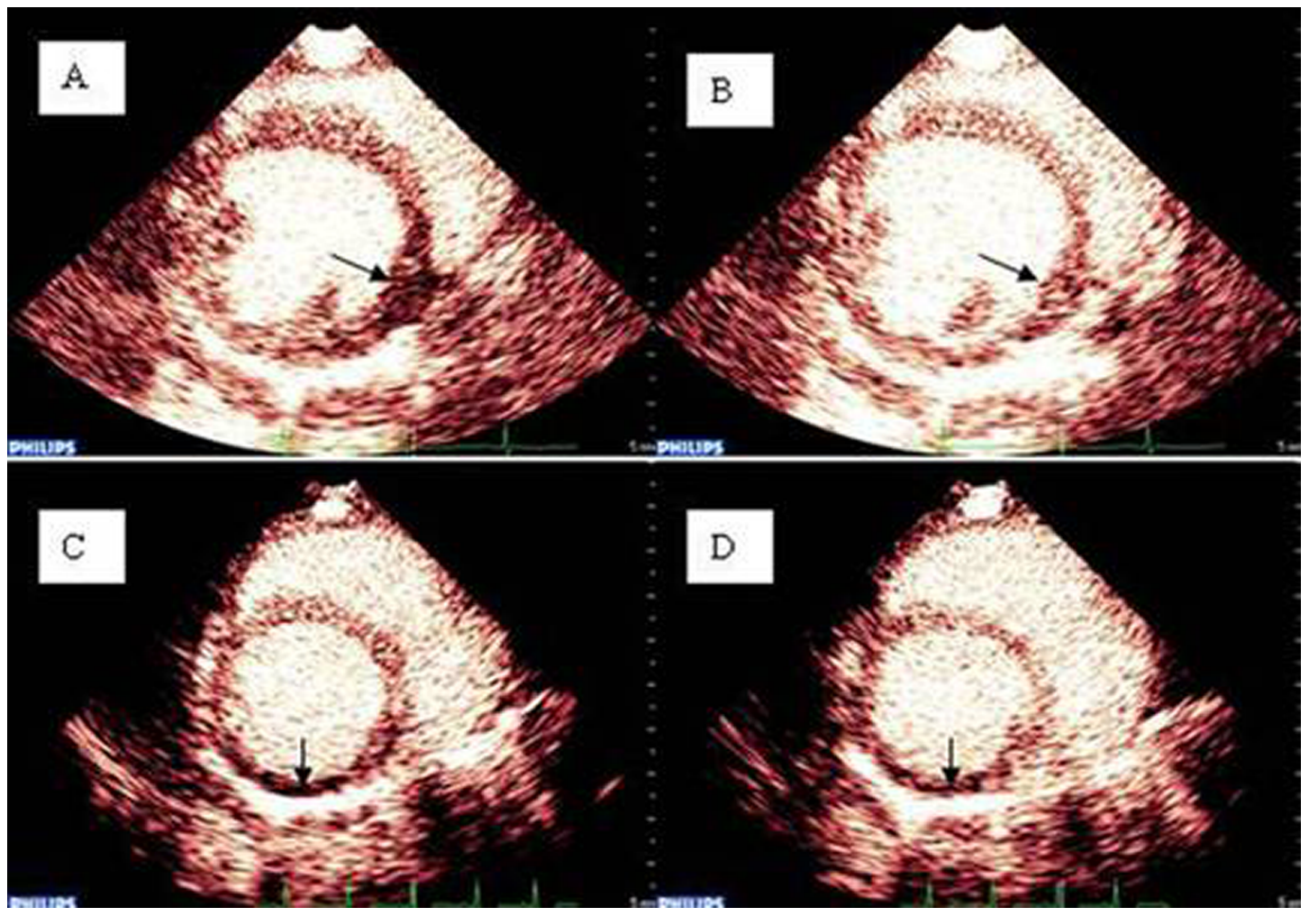
An illustration of RT-MCE between the CRT and control groups. The arrows indicate the ischemic areas. The images in A and B show the improvement of myocardial perfusion before and after bi-ventricular pacing in the CRT group (MBF: 23 dB/S *vs.* 58 dB/s). The images in C and D show no significant improvement of myocardial perfusion in the ischemic area of the control group (MBF: 19 dB/S *vs.* 23 dB/s).

The MBF value in normal beagles was 73.5±10.5 dB/s. No difference was observed between the two groups at baseline (70.2±11.3 dB/s vs. 76.4±9.3 dB/s, *p*>0.05) or at 2 weeks after MI and before CRT (25.9±5.9 dB/s vs. 23.6±5.7 dB/s). However, MBF values increased significantly in the CRT group compared with the control group after 4 weeks of pacing (49.8±15.5 dB/s vs. 28.5±4.6 dB/s, *p*<0.05; Table 1). These results are consistent with CRT-mediated effect of myocardial perfusion in ischemic heart failure (Figure 2).

### Comparisons between responders and non-responders in the CRT group

**Figure 3:**
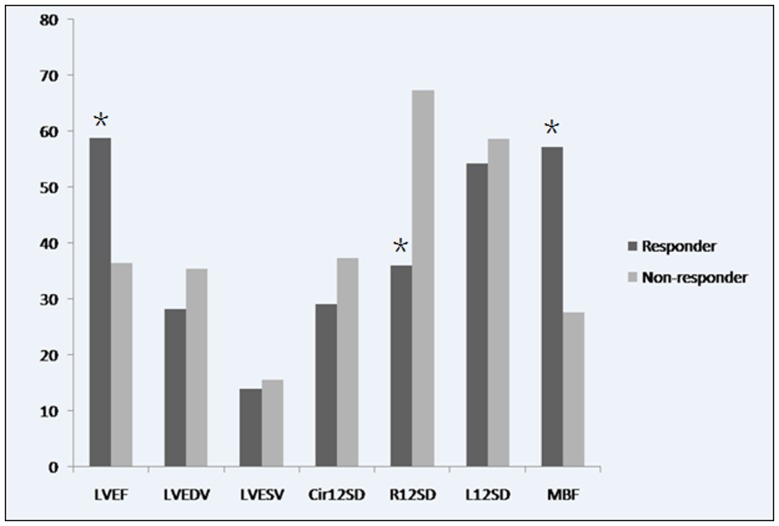
An illustration of LVEF, LVEDV, LVESV, Cir12SD, R12SD, L12SD, and MBF between response group and non-response group. The MBF was significantly improved in response group. LVEF = left ventricular ejection fraction; LVEDV = left ventricular end diastolic volume; LVESV = left ventricular end systolic volume; Cir12SD = standard deviation of the time-to-peak of the transmural regional circumferential strain; R12SD = standard deviation of the time-to-peak of the transmural regional radial strain; L12SD = standard deviation of the time-to-peak of the transmural regional longitudinal strain; MBF = myocardial blood flow.

Since the LVEF of dogs in the control group was spontaneously improved after MI and peaked at 45% by 4 weeks after the intervention, we defined LVEF ≥45% as the cut-off point for distinguishing the responders or non-responders to CRT. Six dogs (75%) in the CRT group were found to be effectively treated by the therapy, the other 2 canine were sorted to as unsuccessful models. Obviously, LVESV and MBF differed significantly between the responders and non-responders before CRT (LVESV: 19.5±3.4 ml vs. 14.4±1.3 ml; MBF: 28.4±4.9 dB/s vs. 19.4±4.3 dB/s, all *p*<0.05), whereas no significant differences in LVEDV, Cir12SD, R12SD, or L12SD were observed (Table 2, Figure 3).

**Table 2:** The echocardiographic parameters between responders and non-responders.

	Response group (n = 6)			Non-response group (n = 2)		
	Baseline	Before CRT	4 w after CRT	Baseline	Before CRT	4 w after CRT
LVEF (%)	71.7±4.2	32.2±4.8	58.8±8.2* †	72.0±2.8	31.0±4.2	36.5±4.9
LVEDV (ml)	26.8±4.4	34.9±2.1	28.2±2.9* †	27.1±0.5	36.7±1.8	35.4±0.9
LVESV (ml)	9.1±5.3	19.5±3.4 †	13.9±2.6*	9.4±2.5	14.4±1.3	15.6±2.4
Cir12SD (ms)	18.4±3.8	40.8±5.8	29.1±6.6*	20.1±0.6	40.6±6.2	37.3±3.1
R12SD (ms)	28.5±5.3	79.9±17.5	36.1±10.7* †	32.0±3.2	72.7±1.7	67.4±0.1
L12SD (ms)	36.2±7.6	56.8±17.9	54.3±10.8	33.8±10.4	67.3±7.4	58.7±7.4
MBF (dB/s)	67.6±12.1	28.4±4.9 †	57.2±8.6* †	77.8±4.0	19.4±4.3	27.6±0.4

*p<0.05 compared to that before CRT;

† *p*<0.05 compared to non-response group.

On the other hand, while the myocardial perfusion was analyzed, the correlation coefficient between MBF before CRT and LVEF in responders after 4 weeks of CRT was 0.917 (*p*<0.05). When MBF>24.9 dB/s was defined as a cut-off value before CRT, the sensitivity and the specificity of predicting the response to CRT were found to be 83.3% and 100%, respectively.

## Discussion

Bi-ventricular pacing has been shown to improve cardiac function, and CRT is an efficient therapeutic choice for patients with heart failure [8]. Most previous studies have focused on the positioning of the pacing electrode, the optimization of the A–V delay, the QRS duration, and the evaluation of LV synchrony [9]–[12]. Meanwhile, effective myocardial perfusion may play an important role in this procedure. To our knowledge, this is the first large animal study which has determined the relationship between myocardial blood circulation evaluated by RT-MCE and the outcome of CRT, in addition, considered the impact of bi-ventricular pacing on myocardial perfusion.

In the first place, our present finding of improved LVEDV in CRT responders after 4 weeks of bi-ventricular pacing, which is consistent with some [13] but not all previous observations [14]. Specifically, Chung et al. recently reported that chronic bi-ventricular pacing in the infarct region fails to improve LVEDV in patients after 1 year follow-up. Interestingly, the LV leads in that particular study were placed in the peri-infarct or infarct area. Indeed, pacing in this region, which is known to exhibit reduced excitability, impaired contractile function, excessive scar formation, and gap junction remodeling, may be suboptimal, potentially explaining the lack of improvement that they observed [15]. Another possible explanation for the divergent outcomes between our present study and those of Chung et al may be related to important differences in the measurements of LVEDV. Chung and colleagues used Biplane, A4C, and A2C to determine LVEDV, whereas we relied on a robust 3D imaging approach acquired from a full volume set of apical four-chamber views which would be more accuracy.

Secondly, 2D-STI is a relatively new technique for the evaluation of prognosis for bi-ventricular pacing. The impact of CRT on longitudinal, radial, and circumferential strains has been documented in multiple studies [16]–[17]. However, the predictive value of these metrics remains controversial. In fact, up to the present, studies addressing the predictive value of LV synchrony in ischemic cardiomyopathy are scarce. Previously, Parsai et al found that CRT response is dictated by normalization of multiple independent mechanisms, of which LV dyssynchrony is only one. They found that long axis dyssynchrony alone failed to detect 40% of responders [18]. And, Gorcsan et al endeavored themselves to combine longitudinal and radial dyssynchrony detection to evaluate the effect of resynchronization therapy [19]. A more recent study showed that CRT resulted in a significant improvement in both LV dyssynchrony and contractile function as measured by global longitudinal strain [20]. In the present study, a significant improvement of radial dyssynchrony after CRT was observed. One potential reason for the divergent outcomes across various studies may be related to key anatomical differences in the occluded coronary arteries, which may have led to differences in the location and size of the MI. For example, whereas the left main and three vessel coronary artery disease were involved in the study by Choi et al [21], only the first diagonal branch was affected in our present study. For all we know, there are few studies addressing the relationship between coronary artery distribution and longitudinal, circumferential, or radial strain. We do believe that further studies are needed to clarify these relationships.

Thirdly, it has been widely accepted that RT-MCE is a non-invasive technique that can be performed at the bedside to assess LV function and coronary flow reserve (CFR) in patients with heart failure [22]. MCE is considered to be a powerful predictor of functional recovery after acute MI [23]–[24]. Recently, Anantharam et al. reported that CFR, calculated as the ratio of MBF during stress to that at rest, is a powerful predictor of mortality in patients with heart failure [22]. Moreover, previous studies indicated that CRT not only improves microcirculatory function [25]–[26] but also increases the left anterior descending coronary artery flow, which is associated with an improvement in regional myocardial contraction [27]. In our study, our findings are clear that MBF at baseline were highly correlated with the efficacy of bi-ventricular treatment and were consistent with CRT induced amendment on myocardial microcirculation. Therefore, as an easy and repeatable means, the evaluation of myocardial perfusion by RT-MCE may be vital to the implementation and optimization of CRT.

In brief, we established an *in vivo* canine model of ischemic cardiomyopathy, in which we demonstrated significant improvement of MBF by CRT. We uncovered important differences in basal (pre-CRT) MBF values between responders and non-responders. The significance of MBF as a predictor of CRT outcome is further highlighted by the fact that no significant differences were observed for the other measured indices of cardiac function, including LVEF, LVEDV, Cir12SD, R12SD, and L12SD. The high degree of correlation between MBF measured before CRT and LVEF measured in the responder subgroup after 4 weeks of CRT is agreed with the notion that MBF may be mechanistically related to CRT success in ischemic cardiomyopathy. In other words, our findings indicate that assessment of myocardial perfusion may be more important than LV dyssynchrony (Cir12SD, R12SD, and L12SD) in selecting appropriate individuals for CRT.

Besides, our findings may also explain the high sensitivity of MBF in detecting hibernating myocardium which has been reported by Fernandes et al. already [28], as well as Hickman et al. The latter concluded that resting MBF could distinguish hibernating myocardium from non-viable myocardium [29]. In our study, MBF after CRT was significantly improved in the responder subgroup, implying that CRT may play a key role in reversing the pathophysiological processes associated with ischemic HF. For example as evidence, Aiba et al. investigated that CRT partially restores ion channel remodeling, abnormal Ca^2+^ homeostasis, and regional heterogeneity of action potential duration induced by the dyssynchronous mechanical activation of the failing LV in a canine model of left bundle branch block coupled with tachypacing induced heart failure [30]. Additionally, Chakir et al. revealed that CRT reverses regional and global molecular remodeling, generating more homogeneous activation of stress kinases and reducing apoptosis in the same model [31]. Moreover, Ashrafian illustrated that neurohumoral activation, including free fatty acid and glucose metabolism as well as insulin resistance, may be involved in the beneficial effects of CRT [32]. Indeed, all of these factors may be mechanistically related to the improvement in MBF by CRT. But, further studies are required to determine the individual contributions of these mechanisms to CRT-mediated changes in MBF.

## Limitations

The present study has some important limitations that require mention. First of all, we relied on a clinically relevant large animal model. Therefore, the number of subjects per group (especially the non-responder subgroup in CRT group) was by necessity limited when compared to rodent studies. In the next place, our study focused on the global dyssynchrony of the left ventricle, was not designed to uncover potentially important differences in intraventricular properties between various segments within the LV. Thirdly, despite its clinical relevance, our model of MI involved ligation of the first diagonal branch, and therefore, future studies in which the severity of MI can be modulated by ligating additional coronary arteries are warranted. Fourthly, as we mentioned, the pathophysiological process of CRT remains unclear. Varies studies are required to determine the cellular and molecular mechanisms that underlie the observations of the present study. Finally, as with any other animal study, our preclinical findings will require validation in humans before they can be appropriately translated to clinical applications.

## Conclusions

The MBF value is positively correlated with the responsiveness to CRT and, therefore, should be considered an important predictor of CRT outcome in the setting of ischemic cardiomyopathy. Meanwhile, we’re pretty sure that CRT can improve both cardiac synchrony and myocardial perfusion in this clinically relevant large animal model of ischemic cardiomyopathy.
